# Hochu-Ekki-To Improves Motor Function in an Amyotrophic Lateral Sclerosis Animal Model

**DOI:** 10.3390/nu11112644

**Published:** 2019-11-04

**Authors:** Mudan Cai, Eun Jin Yang

**Affiliations:** 1 Department of Herbal medicine Research, Korea Institute of Oriental Medicine, 1672 Yuseong-daero, Yuseong-gu, Daejeon 305-811, Korea; mudan126@kiom.re.kr; 2 Department of Clinical Research, Korea Institute of Oriental Medicine, 1672 Yuseong-daero, Yuseong-gu, Daejeon 305-811, Korea

**Keywords:** amyotrophic lateral sclerosis, Hochu-ekki-to, herbal medicine, muscle dysfunction, motor neuronal cell death

## Abstract

Hochu-ekki-to (Bojungikgi-Tang (BJIGT) in Korea; Bu-Zhong-Yi-Qi Tang in Chinese), a traditional herbal prescription, has been widely used in Asia. Hochu-ekki-to (HET) is used to enhance the immune system in respiratory disorders, improve the nutritional status associated with chronic diseases, enhance the mucosal immune system, and improve learning and memory. Amyotrophic lateral sclerosis (ALS) is pathologically characterized by motor neuron cell death and muscle paralysis, and is an adult-onset motor neuron disease. Several pathological mechanisms of ALS have been reported by clinical and *in vitro*/*in vivo* studies using ALS models. However, the underlying mechanisms remain elusive, and the critical pathological target needs to be identified before effective drugs can be developed for patients with ALS. Since ALS is a disease involving both motor neuron death and skeletal muscle paralysis, suitable therapy with optimal treatment effects would involve a motor neuron target combined with a skeletal muscle target. Herbal medicine is effective for complex diseases because it consists of multiple components for multiple targets. Therefore, we investigated the effect of the herbal medicine HET on motor function and survival in hSOD1^G93A^ transgenic mice. HET was orally administered once a day for 6 weeks from the age of 2 months (the pre-symptomatic stage) of hSOD1^G93A^ transgenic mice. We used the rota-rod test and foot printing test to examine motor activity, and Western blotting and H&E staining for evaluation of the effects of HET in the gastrocnemius muscle and lumbar (L4–5) spinal cord of mice. We found that HET treatment dramatically inhibited inflammation and oxidative stress both in the spinal cord and gastrocnemius of hSOD1^G93A^ transgenic mice. Furthermore, HET treatment improved motor function and extended the survival of hSOD1^G93A^ transgenic mice. Our findings suggest that HET treatment may modulate the immune reaction in muscles and neurons to delay disease progression in a model of ALS.

## 1. Introduction

Amyotrophic lateral sclerosis (ALS), also known as Lou Gehrig’s disease, is characterized by a loss of motor neurons, muscle weakness, and spasticity [1]. ALS can be divided into familiar ALS (fALS), which is caused by autosomal dominant mutations in genes such as superoxide dismutase (SOD)1, and sporadic ALS (sALS). However, some gene mutations have been found to be involved in both fALS and sALS, including mutations of TAR DNA-binding protein (TDP) 43, fused in sarcoma (FUS), valosin-containing protein (VCP), and TATA-binding protein-associated factor 15 (TAF15) [2].

Several pathological mechanisms underlying ALS have been reported, including proteasome and autophagy dysfunction, ER stress, oxidative stress, and mitochondrial disorders [3]. Most notably, a dysregulated immune response plays a critical role in disease progression, as revealed by both ALS animal model and clinical studies [4,5,6]. 

In the central nervous system (CNS), neuroinflammation that is mediated by microglia is involved in the pathogenesis of neurodegenerative diseases such as Alzheimer’s Disease (AD), Parkinson’s Disease (PD), and ALS. In ALS, specific gene mutations in the CNS have been found to contribute to immune dysfunction, including mutations of SOD1, TARDBP, and C9orf72 [7,8,9]. A mutant SOD1 overexpressed animal model was found to exhibit motor neuron dysfunction that was induced by an increase in activated microglia in the peripheral nervous system and CNS [10]. In addition, the expression of IL-6 has been reported to increase via activation of microglia and macrophages in both an animal model of and patients with ALS [11,12]. In the muscles, alternation of neuromuscular junction (NMJ) and muscle denervation that involves a loss of presynaptic terminals, Schwann cells, and axonal degeneration, has been found to lead to clinical weakness and an increased disease severity in patients with ALS [13]. Furthermore, activated macrophages reportedly surround NMJs in symptomatic and end-stage mouse models of ALS [14], and complement factors are upregulated to recruit macrophages in the denervated muscle of a SOD1^G93A^ mouse model [15]. Therefore, immune enhancers could be a candidate for attenuating disease progression and enhancing homeostasis of the body in patients with ALS. 

Herbal medicine has been widely used in Asian countries for thousands of years because of antinociceptive, analgesic, and anti-inflammatory effects, both centrally and peripherally [16,17]. Simply put, herbal medicine can stimulate the immune system and maintain the internal balance of the body. In the case of AD, bioactive components from herbal medicines such as Radix Polygalae, *Panax ginseng*, and *Ginko biloba* have been shown to effectively improve AD symptoms by targeting autophagy [18]. In ALS, many experimental studies have demonstrated that Chinese prescriptions have anti-inflammatory and anti-oxidant effects. In patients with ALS, Chinese prescriptions, including Jiawei Sijunzi, and Dihuang Yinzi, have been found to improve phenotype symptoms and functional rating scales [19,20]. However, further evidence for the efficacy, mechanisms of action, and safety of herbal medicines in the treatment of ALS is required. 

Hochu-ekki-to (HET) in Japanese herbal (Kampo) medicine is similar to Bojungikgi-Tang (BJIGT) in Korea and Bu-Zhong-Yi-Qi Tang in Chinese medicine. HET has ten component herbs, as follows: Astragali radix (16.7%, *A. membranaceus Bunge*), Atractylodes lancea Rhizome (16.7%, rhizomes of *A*. *lancea* DC.), Ginseng radix (16.7%, *P. ginseng* C.A. Meyer), Angelica Radix (12.5%, *Angelica acutiloba* Kitagawa), Bupleuri radix (8.3%, *Bupleurum falcatum* L.), Zizyphi fructus (8.3%, *Zizyphus jujuba* Miller var. inermis Rehder), Aurantii nobilis pericarpium (8.3%, *Citrus unshu* Markovich), Glycyrrhizae radix (6.3%, *Glycyrrhiza uralensis* Fisch et DC.), Cimicifugae Rhizoma (4.2%, *Cimicifuga simplex* Worms kjord), and Zingiberis Rhizoma (2%, *Zingiber officinale* Roscoe) and it was provided by Tsumura pharmaceutical company [21,22]. In addition, Dan et al., and Yae et al., had already reported chemical profile of HET by 3-dimensional HPLC.

HET has been used to enhance the immune system in respiratory disorders [23,24] and to improve the nutritional status associated with chronic diseases [25]. Thus, many studies have investigated the immunopharmacological activities of HET [26,27,28]. In addition, Kiyohara et al. reported that HET enhanced the mucosal immune system [29]. Shih et al. found that HET improved learning and memory, and had an anti-aging effects in a senescence-accelerated mouse model [29]. Furthermore, the authors suggested that HET can penetrate the blood–brain barrier by increasing dopamine and noradrenaline levels in the brain. 

ALS causes both motor neuron death and skeletal muscle paralysis. A suitable therapy with optimal treatment effects for patients with ALS would involve a motor neuron target combined with a skeletal muscle target. In this sense, herbal medicine is effective for complex disease because herbal medicine consists of multiple components. Therefore, we investigated the effect of HET on neuroinflammation, motor function, and muscle weakness in a hSOD1^G93A^ animal model.

## 2. Materials and Methods

### 2.1. Animals

Male hemizygous hSOD1^G93A^ transgenic mice and female B6SJL mice were purchased from the Jackson Laboratory (Bar Harbor, ME, USA) and maintained as described previously [30]. hSOD1^G93A^ mice have a glycine-to-alanine base-pair mutation at the 93rd codon of the cytosolic Cu/Zn superoxide dismutase gene. Male hSOD1^G93A^ mice were housed at 3–4 per cage under specific pathogen-free conditions and had *ad libitum* access to food and water. The facilities were maintained under a constant temperature (21 ± 3 °C) and humidity (50 ± 10%) with a 12 hours light/dark cycle (lights on 07:00–19:00). All mice were treated in accordance with the animal care guidelines of the Korea Institute of Oriental Medicine (protocol number: 13–109). 

### 2.2. Hochu-Ekki-To (HET) Treatment

**Figure 1:**
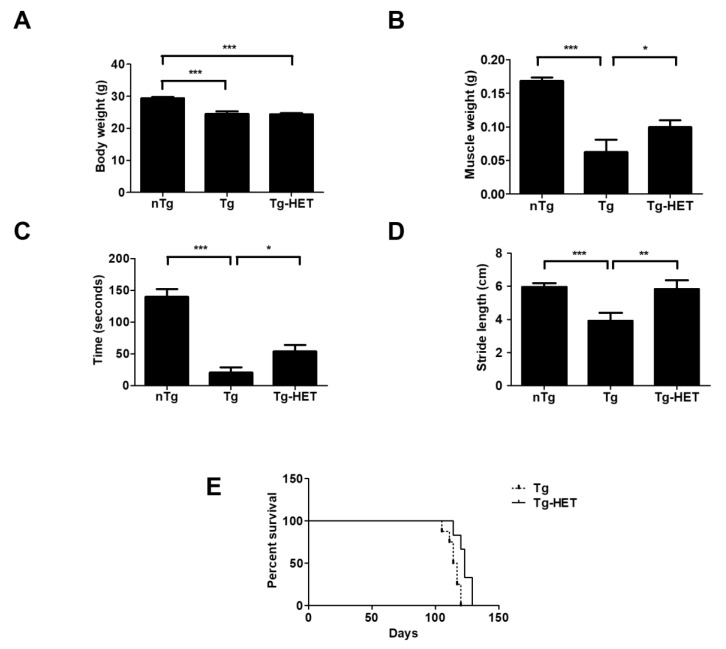
Hochu-ekki-to (HET) treatment ameliorates motor activity and prolongs the life span of a mouse model of amyotrophic lateral sclerosis (ALS). HET (1 mg/g) was orally administered once a day for 6 weeks from the age of 2 months. (**A**) Comparison of body weight between the nTg, Tg, and HET-treated Tg groups. (**B**) Comparison of gastrocnemius weight between the nTg, Tg, and HET-treated Tg groups. (**C**) Motor function was measured by the rota-rod test in all groups. (**D**) The representative average of stride length (*n* = 7/group) of each group, measured using the foot print test. (**E**) Survival rate was calculated by Kaplan-Meyer analysis in Tg and HET-treated Tg (*n* = 8/group). Data are shown as the mean ± SEM. * *p* < 0.05, ** *p* < 0.01, *** *p* < 0.001. nTg: non-transgenic mice, Tg: hSOD1^G93A^, Tg-HET:HET-treated hSOD1^G93A^.

Hochu-ekki-to (HET) was purchased from TSUMURA Co. Ltd (TSUMURA, Osaka, Japan) and diluted at 1 mg/g with autoclaved distilled water. The mice were randomly divided into three groups, as follows: a non-transgenic mice group (nTg, *n* = 8), a hSOD1^G93A^ transgenic mice group (Tg, *n* = 11), and a HET treated hSOD1^G93A^ transgenic mice group (Tg-HET, *n* = 11) (Figure 1). HET (1 mg/g) was orally administered with a disposable oral gavage syringe (FUCHIGAMI, Kurume, Japan) once a day for 6 weeks from the age of 2 months (the pre-symptomatic stage). The dose was translated from human to animal based on a previous study [31].

### 2.3. Rota-Rod Test

The rota-rod test is used to assess motor activity and balance in rodents. Mice were trained every other day for 2 weeks to adapt to the apparatus (Rotarod, B.S Technolab Inc., Korea). During training, the rota-rod was maintained at a constant speed of 10 rpm for 180 seconds. After the last administration of HET, mice performed the test, and we recorded the time mice remained on the rod before falling. Each mouse performed three trials and the average time spent on the rod was determined for each group.

### 2.4. Foot Print Test

The day before mice were sacrificed, the footprint test was used to measure gait. To record stride length, mice hind paws were stained with nontoxic water-soluble black ink, and the alley floor (70 cm length, 6 cm width, and 16 cm height) was covered with white paper to absorb the ink. Each mouse performed three trials and the average of stride length was determined for each group.

### 2.5. Survival Test

To measure lifespan, male transgenic mice were randomly divided into the following treatment groups: distilled water-treated ALS mice (*n* = 8) and ALS mice treated with HET for 6 weeks (*n* = 8/group). Death was defined according to our previous paper [32]. 

### 2.6. Tissue Preparation 

Body weight of mice was measured and mice were anesthetized using pentobarbital sodium (Entobar, Hanlim Pharm, Co., Ltd., Seoul, Korea) and perfused with phosphate-buffered saline (PBS). The gastrocnemius muscle and spinal cord of the mice were dissected and stored at −80 °C until use. The gastrocnemius muscle weight was recorded and the average value for each group recorded. For hematoxylin and eosin (H&E) staining, the gastrocnemius muscle of the mice was fixed in 4% paraformaldehyde at 4 °C before embedding in paraffin. The tissues were cut into transverse sections (5 μm thick) using a microtome (Leica biosystems, IL, USA) and mounted on glass slides.

### 2.7. Western Blotting

For Western blotting, the gastrocnemius muscle and lumbar (L4–5) spinal cord of mice were homogenized in radioimmunoprecipitation assay buffer (50 mM, Tris-HCl (pH 7.4); 1% Nonidet P−40; 0.1% sodium dodecyl sulfate; 150 mM NaCl) containing protease and a phosphatase inhibitor cocktail (Thermo, Waltham, MA, USA). Homogenized tissues were centrifuged at 20,800 × g for 15 minutes at 4 °C. The protein concentration was determined using the Bicinchoninic Acid Assay Kit (Pierce, IL, USA). The samples (20 μg of protein) were denatured with sodium dodecyl sulfate sampling buffer, separated using SDS-PAGE electrophoresis, and transferred to a Polyvinylidene difluoride membrane (Bio-Rad, Hercules, CA, USA). Membranes were incubated in a blocking solution (5% skim milk in TBS) for 1 hour at room temperature then incubated in the various primary antibodies (anti-iba-1, anti-GFAP, anti-TLR4, anti-BAX, anti-HO1, anti-transferrin, anti-CD11b, anti-Ferritin, anti-tubulin, and anti-actin) overnight at 4 °C. The next day, blots were washed and incubated with horseradish peroxidase-conjugated secondary antibodies, and then visualized using the SuperSignal West Femto Substrate Maximum Sensitivity Substrate (Thermo Fisher Scientific, Waltham, MA, USA). For detection of the other antibodies, membranes were stripped in a stripping buffer (Thermo Fisher Scientific, Waltham, MA, USA). The blots were analyzed using the ChemiDoc imaging system (Bio-Rad, Hercules, CA, USA), which were then quantified using the NIH ImageJ program (National Institutes of Health, Bethesda, MD, USA).

### 2.8. H&E Staining and Immunohistohcemistry

For H&E staining, the paraffin sections were de-paraffinized in xylene and rehydrated in a graded alcohol series (100%, 95%, 80% ethanol), followed by deionized H_2_O. Slices were incubated in hematoxylin (Sigma-Aldrich Corp., St. Louis, MO, USA) for 6 minutes and washed under flowing distilled water for 5 minutes, then incubated in eosin for 45 seconds, dehydrated (95%, 100%, xylene), and mounted using a Histomount medium (Sigma-Aldrich Corp.). Immunohistochemistry was performed with previous paper described [32]. In brief, de-paraffinized slides were incubated with 3% hydrogen peroxide (H_2_O_2_) and 5% bovine serum albumin (BSA) in 0.01% PBS-Triton X–100 (Sigma-Aldrich, Oakville, ON, Canada). The sections were incubated with anti-IL-1β (Abcam, Cambridge, UK) and then secondary antibody. For observation, the ABC kit and 3,3′-diaminobenzidine (DAB)/H_2_O_2_ substrate were used with a hematoxylin counterstain. Immunostained tissues were observed with a light microscope (Olympus, Tokyo, Japan). The central nuclei (as a marker of abnormal nuclei) were counted and expressed as a percentage: the number of myocytes with central nuclei divided by the total number of myocytes in each captured image. For the quantification of myocyte cross-sectional area (CSA), the average area of individual myocytes was measured using the NIH ImageJ program.

### 2.9. Statistical Analysis

All values are expressed as the mean ± SEM. The results were analyzed using a one-way analysis of variance (ANOVA) followed by the Newman-Keuls’s *post hoc* test for multiple comparisons. For survival test, the data were analyzed by Kaplan–Meier survival curves. Data were analyzed using GraphPad Prism 5.0 (GraphPad Software, San Diego, CA, USA). Statistical significance was set at *p* < 0.05.

## 3. Results

### 3.1. Hochu-Ekki-(HET) Extended Survival and Improved Motor Function

To examine the effects of HET on physical function, we measured the body and muscle weight of symptomatic HET-treated hSOD1^G93A^ mice (Tg-HET). As shown in Figure 1A, body weight of hSOD1^G93A^ mice (Tg) was lower than that of age-matched non-Tg (nTg); however, there was no significant difference in body weight between the Tg and Tg-HET groups. HET treatment resulted in a 1.6-fold significant increase in the weight of the gastrocnemius muscle compared to that of Tg mice (Figure 1B). Furthermore, we found that HET treatment resulted in a 2.8-fold improvement in motor function in symptomatic hSOD1^G93A^ mice, as revealed in the rota-rod test (Figure 1C). Motor activity was assessed by measuring stride length through the foot print test. The stride length of Tg-HET mice was 1.5-fold greater than age-matched Tg mice (Figure 1D). Furthermore, HET treatment extended the survival rate compared to that of Tg mice (Figure 1E). These findings suggest that HET treatment can prevent motor neuron death and skeletal muscle paralysis in hSOD1^G93A^ mice. 

### 3.2. Hochu-Ekki-To (HET) Reduces Neuroinflammation and Oxidative Stress in the Spinal Cord of hSOD1^G93A^ Mice

**Figure 2:**
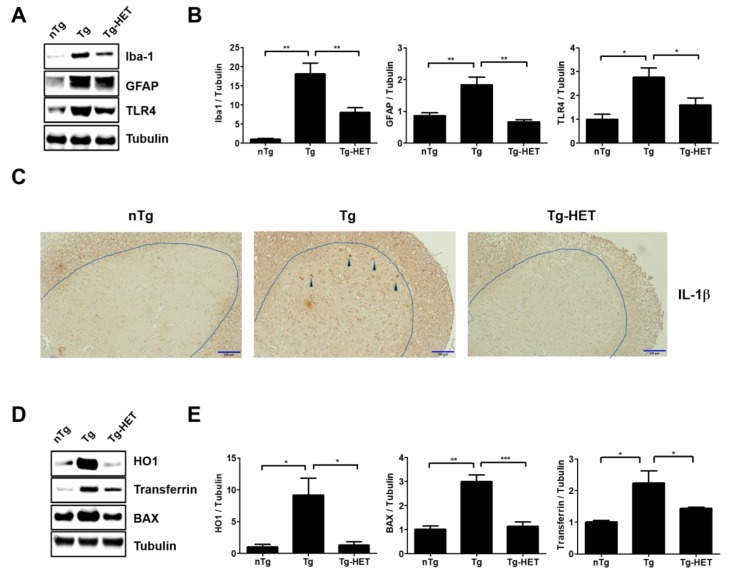
Hochu-ekki-to (HET) increases anti-inflammation and anti-oxidative stress effects in the spinal cord of an ALS mouse model. (**A**) Representative Western blots on inflammatory protein levels of Iba-1 (a marker of microglia), GFAP (a marker of astrocytes), and TLR4 in the spinal cord of each group (nTg, Tg, and Tg- HET). Tubulin was used as a loading control. (**B**) Quantification of the expression level of Iba-1/Tubulin, GFAP/Tubulin, and TLR4/Tubulin in each immunoblot. (**C**) Representative images of IL-1β immunoreatcivity in the anterior horn of the spinal cord in each group. Scale bars = 100 μm (**D**) Representative images of oxidative stress-related proteins (BAX, HO1, and Transferrin) in the spinal cord of each group mice. (**E**) Quantification of the expression levels of BAX/Tubulin, HO1/Tubulin, and transferrin/Tubulin. Data are presented as the mean ± SEM (*n* = 3/group). * *p* < 0.05, ** *p* < 0.01, *** *p* < 0.001. nTg: non-transgenic mice, Tg: hSOD1^G93A^, Tg-HET:HET-treated hSOD1^G93A^.

In our previous study, we found that hSOD1^G93A^ transgenic mice had increased neuroinflammation, indicated by an increase in CD11b, GFAP, Iba-1, and TLR4 (inflammatory proteins in spinal cord) [33,34]. To investigate the effect of HET on neuroinflammation of the spinal cord in hSOD1^G93A^ mice, we investigated the expression of neuroinflammation-related proteins (Iba-1, GFAP, and TLR4) using immunoblotting. As shown in Figure 2A,B, the expression levels of Iba-1, GFAP, and TLR4 in the spinal cord were significantly greater by 18-, 2.1-, and 2.8-fold in symptomatic Tg mice compared to those of nTg mice. However, HET treatment dramatically reduced the levels of Iba-1, GFAP, and TLR4 proteins by 2.3-, 2.7-, and 1.7-fold compared to that of Tg mice. In addition, proinflammatory cytokine, IL-1β immunoreactivity was increased in anterior horn of spinal cord of symptomatic Tg mice, but it was reduced by treatment with HET (Figure 2C). Furthermore, we found evidence for anti-neuroinflammatory effects of HET, and observed a reduction of oxidative stress in the spinal cord of Tg mice. Oxidative stress-related proteins HO1, transferrin, and BAX were significantly lower by 7-, 2.6-, and 1.6-fold in the spinal cord of Tg-HET mice compared to that of age-matched Tg mice (Figure 2D,E). Taken together, HET treatment seems to enhance neuroimmune systems to maintain motor neuron survival and consequently improve motor function in the ALS animal model. 

### 3.3. Hochu-Ekki-To (HET) Attenuates Muscle Dysfunction

**Figure 3:**
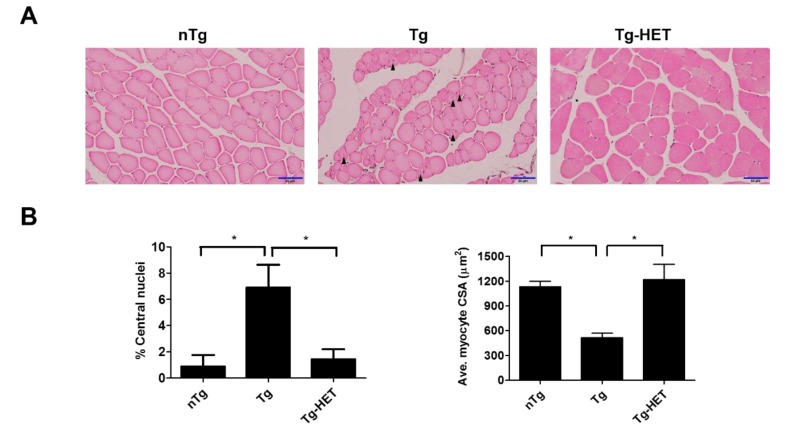
Hochu-ekki-to (HET) treatment has a protective effect against muscle atrophy in the gastrocnemius of an ALS mouse model. (**A**) Representative images of H&E staining showing the muscle atrophy condition, such as smaller myocytes and abnormal nuclei in gastrocnemius of hSOD1^G93A^ mice. Arrowheads indicate abnormal nuclei (central nucleation) in myocytes. (**B**) Abnormal nuclei were expressed as a percentage of abnormal nuclei (left panel). Quantified average myocyte cross-sectional area (CSA) (right panel). Scale bar = 50 μm Data are presented as the mean ± SEM (*n* = 3/group). * *p* < 0.05. nTg: non-transgenic mice, Tg: hSOD1^G93A^, Tg-HET:HET-treated hSOD1^G93A^.

In our previous study, we found that HO1, Transferrin, BAX, and Ferritin (as oxidative stress-related proteins) were increased in the spinal cord of hSOD1^G93A^ mice [35,36]. To examine the effect of HET on the weakness of skeletal muscle during ALS progression, we investigated the expression level of inflammatory and oxidative stress-related proteins in the gastrocnemius muscle of symptomatic hSOD1^G93A^ mice. The smaller myocytes with abnormal nuclei that had moved to the center of the cells in the gastrocnemius of hSOD1^G93A^ mice. As shown in Figure 3A,B, we found that the percentage of central nuclei was increased by 7.8-fold in the gastrocnemius muscle of symptomatic hSOD1^G93A^ mice compared to nTg mice (Figure 3B). In addition, the average CSA of myocytes was reduced by 2.2-fold in symptomatic hSOD1^G93A^ mice compared to nTg mice (Figure 3B). However, HET treatment led to decrease 4.8-fold in the percentage of central nuclei and increase 2.4-fold the average CSA of myocytes in the gastrocnemius of hSOD1^G93A^ mice.

**Figure 4:**
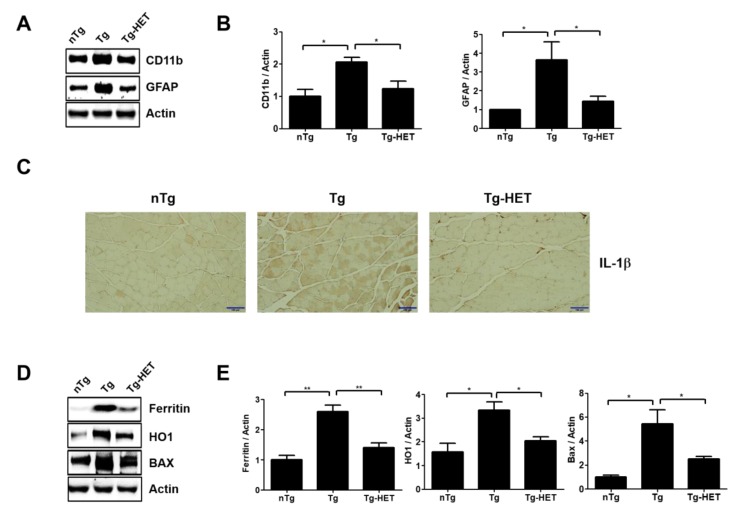
Hochu-ekki-to (HET) treatment enhances anti-inflammation, anti-oxidative stress effects, and regulates autophagy in the gastrocnemius of an ALS mouse model. (**A**) Representative Western blots of inflammatory related proteins, CD11b and GFAP, in the gastrocnemius of each group. Actin was used as a loading control. (**B**) Quantification of the expression levels of CD11b/Actin and GFAP/Actin. (**C**) Representative images of IL-1β immunostaining in the gastrocnemius of each group. Scale bars = 100 μm (**D**) Representative images of oxidative stress-related proteins (Ferratin, HO1, and BAX) in the gastrocnemius of each group. (**E**) Quantification of the expression levels of Ferritin/Actin, HO1/Actin, and BAX/Actin. Data are presented as the mean ± SEM (*n* = 3/group). * *p* < 0.05, ** *p* < 0.01. nTg: non-transgenic mice, Tg: hSOD1^G93A^, Tg-HET:HET-treated hSOD1^G93A^.

In addition, myocyte was small in the gastrocnemius muscle of symptomatic hSOD1^G93A^ mice. However, HET treatment inhibited the muscle atrophy seen in the gastrocnemius by H&E staining (Figure 3). This suggests that HET treatment can reduce muscle damage and inflammation in the gastrocnemius of symptomatic hSOD1^G93A^ mice. To address this hypothesis, we investigated the expression level of inflammatory proteins including CD11b and GFAP and oxidative stress-related proteins such as Ferritin, HO1, and BAX in gastrocnemius of symptomatic hSOD1^G93A^ mice. As expected, HET treatment significantly reduced the expression levels of GFAP and CD11b by 1.7- and 2.5-fold, respectively, in the gastrocnemius of hSOD1^G93A^ mice (Figure 4A,B). In addition, proinflammatory cytokine, IL-1β immunoreactivity was increased in the gastrocnemius of symptomatic Tg mice, but it was reduced by treatment with HET (Figure 4C). Furthermore, HET treatment significantly reduced the levels of Ferritin, HO1, and BAX by 1.9-, 1.6-, and 2.2-fold, respectively, in the gastrocnemius of hSOD1^G93A^ mice (Figure 4D,E). These findings suggest that HET treatment may boost the immune system to protect from muscle loss and damage in this model of ALS. 

## 4. Discussion

ALS is a disease with complex pathological mechanisms and no effective drug treatment. Herbal medicine is composed of multiple components and is used for multi-targets. In addition, herbal medicine focuses on boosting the immune system and maintaining an internal balance of the body. To investigate the possibility of using herbal medicine as treatment for ALS, we investigated the effect of HET treatment on the spinal cord and skeletal muscle in an animal model of ALS. 

Neuroinflammation in the brain occurred via microglial proliferation and astrocytic hypertrophy. Microglia are immune cells in the CNS that play a role in clearing pathogens through phagocytosis and play a critical role in homeostasis [37]. Microglial cell activation increases the expression of inflammatory cytokines such as IL-6 and IL1β and leads to oxidative stress and neuroinflammation, which results in augmented microglial NADPH-derived ROS accumulation [38,39]. In ALS, microglial activation is correlated with neuroinflammation and disease progression [40]. Correspondingly, minocycline treatment has been found to reduce neuroinflammation and microglial activation in clinical trials with patients with ALS [41]. However, it is not effective in patients with ALS who have other neurological disorders. Hence, herbal medicine may be a good, more effective candidate for protecting neurons and skeletal muscle from degeneration, primarily because herbal medicine contains multiple compounds and targets. In this study, HET treatment reduced the expression levels of CD11b and GFAP in the spinal cord and gastrocnemius of symptomatic hSOD1^G93A^ mice. This suggests that HET treatment can enhance the body’s immune system and extend the survival rate of these mice. As expected, we found that HET treatment increases gastrocnemius weight and survival rate of hSOD1^G93A^ mice. 

Oxidative stress and inflammation are significant factors in ALS pathogenesis, and lead to motor neuron death and severe muscle degeneration. While motor neurons control muscle function, retrograde signals can pass from the muscle back to motor neurons via the NMJ [42]. In addition, previous work has found that oxidative stress leading to muscle atrophy was increased in the pre-symptomatic stages in hSOD1^G86R^ mice [43]. In our study, we found that oxidative stress-related proteins such as Ferritin, HO1, Transferrin, and BAX were dramatically increased in the gastrocnemius and the spinal cord of symptomatic hSOD1^G93A^ mice. Furthermore, HET treatment significantly attenuated the expression level of oxidative stress-related proteins in the muscle and spinal cord of hSOD1^G93A^ mice. Patients with ALS have defective energy homeostasis, and skeletal muscle degeneration is a critical factor in the pathogenesis of ALS and its symptoms. Some studies have provided consistent evidence by demonstrating that atrophy occurred before motor neuron loss and neurodegeneration [44,45]. Furthermore, studies with patients with ALS (fALS and sALS) and animal models of ALS (hSOD1^G93A^ and ^G86R^ models) have reported increased energy expenditure and a defective energy balance due to increased oxidative stress, mitochondrial dysfunction, and inflammation [46,47,48].

## 5. Conclusions

In this study, HET treatment improved muscle function and the survival rate via a reduction of inflammation-related events in both the spinal cord and gastrocnemius of symptomatic hSOD1^G93A^ mice. This suggests that HET treatment can be used to boost immune responses and homeostasis in not only ALS, but also other neurodegenerative diseases. Since ALS is a heterogeneous disease, our findings of a protective effect of HET against muscle atrophy should be verified using other genetic mutation models involving ALS mice of both sexes. Furthermore, patients with ALS have a diverse range of pathologies compared to hSOD1^G93A^ mice. Therefore, future work could examine tissue or cells from patients with ALS treated with HET. Another future challenge would be to identify the bioactive compound of HET, which is composed of ten herbs, to pinpoint the specific molecular mechanisms underlying the positive effects of this herbal medicine. 

## References

[1] Orsini M., Oliveira A.B., Nascimento O.J., Reis C.H., Leite M.A., de Souza J.A., Pupe C., de Souza O.G., Bastos V.H., de Freitas M.R. (2015). Amyotrophic Lateral Sclerosis: New Perpectives and Update. Neurol. Int..

[2] Robberecht W., Philips T. (2013). The changing scene of amyotrophic lateral sclerosis. Nat. Rev. Neurosci..

[3] Mesika R., Reichmann D. (2019). When safeguarding goes wrong: Impact of oxidative stress on protein homeostasis in health and neurodegenerative disorders. Adv. Protein Chem. Struct. Biol..

[4] Rusconi M., Gerardi F., Santus W., Lizio A., Sansone V.A., Lunetta C., Zanoni I., Granucci F. (2017). Inflammatory role of dendritic cells in Amyotrophic Lateral Sclerosis revealed by an analysis of patients’ peripheral blood. Sci. Rep..

[5] McCauley M.E., Baloh R.H. (2019). Inflammation in ALS/FTD pathogenesis. Acta. Neuropathol..

[6] Lyon M.S., Wosiski-Kuhn M., Gillespie R., Caress J., Milligan C. (2019). Inflammation, Immunity, and amyotrophic lateral sclerosis: I. Etiology and pathology. Muscle Nerve.

[7] Rosen D.R., Siddique T., Patterson D., Figlewicz D.A., Sapp P., Hentati A., Donaldson D., Goto J., O’Regan J.P., Deng H.X. (1993). Mutations in Cu/Zn superoxide dismutase gene are associated with familial amyotrophic lateral sclerosis. Nature.

[8] Neumann M., Sampathu D.M., Kwong L.K., Truax A.C., Micsenyi M.C., Chou T.T., Bruce J., Schuck T., Grossman M., Clark C.M. (2006). Ubiquitinated TDP-43 in frontotemporal lobar degeneration and amyotrophic lateral sclerosis. Science.

[9] DeJesus-Hernandez M., Mackenzie I.R., Boeve B.F., Boxer A.L., Baker M., Rutherford N.J., Nicholson A.M., Finch N.A., Flynn H., Adamson J. (2011). Expanded GGGGCC hexanucleotide repeat in noncoding region of C9ORF72 causes chromosome 9p-linked FTD and ALS. Neuron.

[10] Boillee S., Yamanaka K., Lobsiger C.S., Copeland N.G., Jenkins N.A., Kassiotis G., Kollias G., Cleveland D.W. (2006). Onset and progression in inherited ALS determined by motor neurons and microglia. Science.

[11] Zhao W., Beers D.R., Hooten K.G., Sieglaff D.H., Zhang A., Kalyana-Sundaram S., Traini C.M., Halsey W.S., Hughes A.M., Sathe G.M. (2017). Characterization of Gene Expression Phenotype in Amyotrophic Lateral Sclerosis Monocytes. JAMA Neurol..

[12] Patin F., Baranek T., Vourc’h P., Nadal-Desbarats L., Goossens J.F., Marouillat S., Dessein A.F., Descat A., Hounoum B.M., Bruno C. (2016). Combined Metabolomics and Transcriptomics Approaches to Assess the IL-6 Blockade as a Therapeutic of ALS: Deleterious Alteration of Lipid Metabolism. Neurotherapeutics.

[13] Arbour D., Vande Velde C., Robitaille R. (2017). New perspectives on amyotrophic lateral sclerosis: The role of glial cells at the neuromuscular junction. J. Physiol..

[14] Nardo G., Trolese M.C., de Vito G., Cecchi R., Riva N., Dina G., Heath P.R., Quattrini A., Shaw P.J., Piazza V. (2016). Immune response in peripheral axons delays disease progression in SOD1(G93A) mice. J. Neuroinflammation.

[15] Heurich B., El Idrissi N.B., Donev R.M., Petri S., Claus P., Neal J., Morgan B.P., Ramaglia V. (2011). Complement upregulation and activation on motor neurons and neuromuscular junction in the SOD1 G93A mouse model of familial amyotrophic lateral sclerosis. J. Neuroimmunol..

[16] Almeida R.N., Navarro D.S., Barbosa-Filho J.M. (2001). Plants with central analgesic activity. Phytomedicine.

[17] Bahmani M., Shirzad H., Majlesi M., Shahinfard N., Rafieian-Kopaei M. (2014). A review study on analgesic applications of Iranian medicinal plants. Asian Pac. J. Trop. Med..

[18] Zeng Q., Siu W., Li L., Jin Y., Liang S., Cao M., Ma M., Wu Z. (2019). Autophagy in Alzheimer’s disease and promising modulatory effects of herbal medicine. Exp. Gerontol..

[19] Qiu H., Li J.H., Yin S.B., Ke J.Q., Qiu C.L., Zheng G.Q. (2016). Dihuang Yinzi, a Classical Chinese Herbal Prescription, for Amyotrophic Lateral Sclerosis: A 12-Year Follow-up Case Report. Medicine (Baltimore).

[20] Pan W., Su X., Bao J., Wang J., Zhu J., Cai D., Yu L., Zhou H. (2013). Open Randomized Clinical Trial on JWSJZ Decoction for the Treatment of ALS Patients. Evid. Based Complement Alternat. Med..

[21] Yae S., Takahashi F., Yae T., Yamaguchi T., Tsukada R., Koike K., Minakata K., Murakami A., Nurwidya F., Kato M. (2012). Hochuekkito (TJ-41), a Kampo Formula, Ameliorates Cachexia Induced by Colon 26 Adenocarcinoma in Mice. Evid. Based Complement Alternat. Med..

[22] Dan K., Akiyoshi H., Munakata K., Hasegawa H., Watanabe K. (2013). A Kampo (traditional Japanese herbal) medicine, Hochuekkito, pretreatment in mice prevented influenza virus replication accompanied with GM-CSF expression and increase in several defensin mRNA levels. Pharmacology.

[23] Yang S.H., Kao T.I., Chiang B.L., Chen H.Y., Chen K.H., Chen J.L. (2015). Immune-modulatory effects of bu-zhong-yi-qi-tang in ovalbumin-induced murine model of allergic asthma. PLoS ONE.

[24] Liu L., Hu L., Yao Z., Qin Z., Idehara M., Dai Y., Kiyohara H., Yamada H., Yao X. (2019). Mucosal immunomodulatory evaluation and chemical profile elucidation of a classical traditional Chinese formula, Bu-Zhong-Yi-Qi-Tang. J. Ethnopharmacol..

[25] Tatsumi K., Shinozuka N., Nakayama K., Sekiya N., Kuriyama T., Fukuchi Y. (2009). Hochuekkito improves systemic inflammation and nutritional status in elderly patients with chronic obstructive pulmonary disease. J. Am. Geriatr. Soc..

[26] Utsuyama M., Seidlar H., Kitagawa M., Hirokawa K. (2001). Immunological restoration and anti-tumor effect by Japanese herbal medicine in aged mice. Mech. Ageing Dev..

[27] Suzuki T., Takano I., Nagai F., Fujitani T., Ushiyama K., Okubo T., Seto T., Ikeda S., Kano I. (1999). Suppressive effects of Hochu-ekki-to, a traditional Chinese medicine, on IgE production and histamine release in mice immunized with ovalbumin. Biol. Pharm. Bull..

[28] Kaneko M., Kawakita T., Yamaoka Y., Nomoto K. (2001). Development of the susceptibility to oral tolerance induction in infant mice administered a herbal drug, Hochu-ekki-to (Bu-Zhong-Yi-Qi-Tang). Int. Immunopharmacol..

[29] Kiyohara H., Nagai T., Munakata K., Nonaka K., Hanawa T., Kim S.J., Yamada H. (2006). Stimulating effect of Japanese herbal (kampo) medicine, hochuekkito on upper respiratory mucosal immune system. Evid. Based Complement Alternat. Med..

[30] Jiang J.H., Yang E.J., Baek M.G., Kim S.H., Lee S.M., Choi S.M. (2011). Anti-inflammatory effects of electroacupuncture in the respiratory system of a symptomatic amyotrophic lateral sclerosis animal model. Neurodegener. Dis..

[31] Reagan-Shaw S., Nihal M., Ahmad N. (2008). Dose translation from animal to human studies revisited. FASEB J..

[32] Cai M., Lee S.H., Yang E.J. (2019). Bojungikgi-tang Improves Muscle and Spinal Cord Function in an Amyotrophic Lateral Sclerosis Model. Mol. Neurobiol..

[33] Yang E.J., Jiang J.H., Lee S.M., Yang S.C., Hwang H.S., Lee M.S., Choi S.M. (2010). Bee venom attenuates neuroinflammatory events and extends survival in amyotrophic lateral sclerosis models. J. Neuroinflammation.

[34] Cai M., Choi S.M., Yang E.J. (2015). The effects of bee venom acupuncture on the central nervous system and muscle in an animal hSOD1G93A mutant. Toxins (Basel).

[35] Lee S.H., Yang E.J. (2019). Anti-Neuroinflammatory Effect of Jaeumganghwa-Tang in an Animal Model of Amyotrophic Lateral Sclerosis. Evid. Based Complement Alternat. Med..

[36] Cai M., Yang E.J. (2018). Gamisoyo-San Ameliorates Neuroinflammation in the Spinal Cord of hSOD1(G93A) Transgenic Mice. Mediators Inflamm..

[37] Schafer D.P., Stevens B. (2015). Microglia Function in Central Nervous System Development and Plasticity. Cold Spring Harb. Perspect. Biol..

[38] Weiss A., Attisano L. (2013). The TGFbeta superfamily signaling pathway. Wiley Interdiscip. Rev. Dev. Biol..

[39] Zuroff L., Daley D., Black K.L., Koronyo-Hamaoui M. (2017). Clearance of cerebral Abeta in Alzheimer’s disease: Reassessing the role of microglia and monocytes. Cell Mol. Life Sci..

[40] Henkel J.S., Beers D.R., Zhao W., Appel S.H. (2009). Microglia in ALS: The good, the bad, and the resting. J. Neuroimmune Pharmacol..

[41] Gordon P.H., Moore D.H., Miller R.G., Florence J.M., Verheijde J.L., Doorish C., Hilton J.F., Spitalny G.M., MacArthur R.B., Mitsumoto H. (2007). Efficacy of minocycline in patients with amyotrophic lateral sclerosis: A phase III randomised trial. Lancet Neurol..

[42] Nguyen Q.T., Son Y.J., Sanes J.R., Lichtman J.W. (2000). Nerve terminals form but fail to mature when postsynaptic differentiation is blocked: In vivo analysis using mammalian nerve-muscle chimeras. J. Neurosci..

[43] Halter B., Gonzalez de Aguilar J.L., Rene F., Petri S., Fricker B., Echaniz-Laguna A., Dupuis L., Larmet Y., Loeffler J.P. (2010). Oxidative stress in skeletal muscle stimulates early expression of Rad in a mouse model of amyotrophic lateral sclerosis. Free Radic. Biol. Med..

[44] Dadon-Nachum M., Melamed E., Offen D. (2011). The “dying-back” phenomenon of motor neurons in ALS. J. Mol. Neurosci..

[45] Marcuzzo S., Zucca I., Mastropietro A., de Rosbo N.K., Cavalcante P., Tartari S., Bonanno S., Preite L., Mantegazza R., Bernasconi P. (2011). Hind limb muscle atrophy precedes cerebral neuronal degeneration in G93A-SOD1 mouse model of amyotrophic lateral sclerosis: A longitudinal MRI study. Exp. Neurol..

[46] Dupuis L., Pradat P.F., Ludolph A.C., Loeffler J.P. (2011). Energy metabolism in amyotrophic lateral sclerosis. Lancet Neurol..

[47] Pi-Sunyer F.X. (2000). Overnutrition and undernutrition as modifiers of metabolic processes in disease states. Am. J. Clin. Nutr..

[48] Dupuis L., Oudart H., Rene F., Gonzalez de Aguilar J.L., Loeffler J.P. (2004). Evidence for defective energy homeostasis in amyotrophic lateral sclerosis: Benefit of a high-energy diet in a transgenic mouse model. Proc. Natl. Acad. Sci. USA.

